# Automated inversion time selection for late gadolinium–enhanced cardiac magnetic resonance imaging

**DOI:** 10.1007/s00330-024-10630-w

**Published:** 2024-02-10

**Authors:** Cheng Xie, Rory Zhang, Sebastian Mensink, Rahul Gandharva, Mustafa Awni, Hester Lim, Stefan E. Kachel, Ernest Cheung, Richard Crawley, Leonid Churilov, Nuno Bettencourt, Amedeo Chiribiri, Cian M. Scannell, Ruth P. Lim

**Affiliations:** 1 Melbourne Bioinnovation Student Initiative (MBSI), Parkville, VIC Australia; 2 Department of Radiology, Artificial Intelligence in Radiology Laboratory, Austin Health, 145 Studley Rd, Heidelberg, VIC 3084 Australia; 3 Melbourne Medical School, The University of Melbourne, Parkville, VIC Australia; 4 King’s College London, Strand, London, UK; 5 University of Porto, Porto, Portugal; 6 Eindhoven University of Technology, Eindhoven, the Netherlands

**Keywords:** Magnetic resonance imaging, Myocardium, Deep learning, Neural Networks (Computer), Image Processing (Computer-Assisted)

## Abstract

**Objectives:**

To develop and share a deep learning method that can accurately identify optimal inversion time (TI) from multi-vendor, multi-institutional and multi-field strength inversion scout (TI scout) sequences for late gadolinium enhancement cardiac MRI.

**Materials and methods:**

Retrospective multicentre study conducted on 1136 1.5-T and 3-T cardiac MRI examinations from four centres and three scanner vendors. Deep learning models, comprising a convolutional neural network (CNN) that provides input to a long short-term memory (LSTM) network, were trained on TI scout pixel data from centres 1 to 3 to identify optimal TI, using ground truth annotations by two readers. Accuracy within 50 ms, mean absolute error (MAE), Lin’s concordance coefficient (LCCC) and reduced major axis regression (RMAR) were used to select the best model from validation results, and applied to holdout test data. Robustness of the best-performing model was also tested on imaging data from centre 4.

**Results:**

The best model (SE-ResNet18-LSTM) produced accuracy of 96.1%, MAE 22.9 ms and LCCC 0.47 compared to ground truth on the holdout test set and accuracy of 97.3%, MAE 15.2 ms and LCCC 0.64 when tested on unseen external (centre 4) data. Differences in vendor performance were observed, with greatest accuracy for the most commonly represented vendor in the training data.

**Conclusion:**

A deep learning model was developed that can identify optimal inversion time from TI scout images on multi-vendor data with high accuracy, including on previously unseen external data. We make this model available to the scientific community for further assessment or development.

**Clinical relevance statement:**

A robust automated inversion time selection tool for late gadolinium–enhanced imaging allows for reproducible and efficient cross-vendor inversion time selection.

**Key Points:**

*• A model comprising convolutional and recurrent neural networks was developed to extract optimal TI from TI scout images.*

*• Model accuracy within 50 ms of ground truth on multi-vendor holdout and external data of 96.1% and 97.3% respectively was achieved.*

*• This model could improve workflow efficiency and standardise optimal TI selection for consistent LGE imaging.*

**Supplementary Information:**

The online version contains supplementary material available at 10.1007/s00330-024-10630-w.

## Introduction

Late gadolinium–enhanced (LGE) imaging is integral to clinical cardiac MRI, with applications including evaluation of tissue viability in coronary artery disease and diagnosis of non-ischaemic cardiomyopathies [1, 2]. LGE relies upon appropriate selection of an optimal inversion time (TI) to suppress background myocardial signal [2–4], particularly where only magnitude reconstructed images are available. A Look–Locker TI scout sequence is typically used [5, 6]. The optimal TI varies individually and depends upon contrast agent, dose, acquisition timing and patient factors. Myocardial pathologies impact contrast distribution within the myocardium, for example with increased extracellular volume as is seen in cardiac amyloid, or breach of the intracellular space with myocardial infarction [7]. Whilst phase-sensitive inversion recovery imaging can be performed to minimise dependence upon optimal TI selection [8], TI scout imaging remains widely performed in clinical practice [2].

The optimal TI is typically manually selected by the examining technologist or supervising clinician, subject to factors including level of experience. An automated approach may offer a reproducible, objective and time-efficient alternative. Deep learning methodologies, particularly convolutional neural networks (CNN [9]), have shown promising results in cardiac MRI imaging applications including quantification of LV (left ventricular) function [10], characterisation of tissue scarring [11] and prognostication [12]. Given the spatiotemporal nature of TI scout imaging, an additional recurrent neural network (RNN) may aid in capturing temporal elements [13].

Bahrami et al previously created a deep learning (DL) model for optimal TI selection with promising results in a single-vendor, single-institution setting [14]. However, whether this can translate to a model that performs well on imaging from different institutions is unknown, with TI scout imaging acquired with different pulse sequences and at different field strengths. We hypothesise an integrated CNN and LSTM [15] model which takes advantage of spatiotemporal relations and uses techniques to minimise overfitting can overcome challenges related to generalisability across vendors, institutions and field strengths.

The purpose of our study is to develop an artificial intelligence model capable of selecting optimal TI from scout sequences, applicable to imaging obtained from different institutions and different scanner systems. In addition, we aim to share our developed model with the research community.

## Materials and methods

### Data source

This was an international retrospective cross-sectional study from four centres with institutional ethics approval. The dataset comprised 1136 de-identified studies where TI scout sequences were performed in 1100 patients (mean age 54.5 years, 691 male, 409 female). Studies were performed at 1.5 T or 3 T, with three vendor systems. Thirty-six repeat studies were present in the centre 4 group, not used in model training. The most common indication for centre 1–3 examinations was assessment of scar and myocardial viability (*n* = 259). For centre 4, aortic stenosis was the predominant study indication (54/67, 80.6%). Magnet systems, administered contrast and patient demographics are summarised in Table 1. Scanner-specific acquisition parameters are provided in Supplementary Materials.

**Table 1:** Scan systems, contrast agent used and patient characteristics for each centre

	Centre 1		Centre 2			Centre 3	Centre 4		Total
Scanner vendor and model	Siemens Avanto	Siemens Skyra	Philips Ingenia	Philips Achieva	Siemens Aera	GE Optima MR450w	Siemens Symphony	Philips Achieva	
Magnetic field strength (T)	1.5	3	1.5	3	1.5	1.5	1.5	3	
Contrast agent and dose	Gadoterate meglumine* 0.2 mmol/kg		Gadobutrol** 0.2 mmol/kg			Gadoterate meglumine* 0.2 mmol/kg to maximal volume of 30 mL	Gadobutrol** 0.2 mmol/kg		
Mean age (year) ± SD	53.86 ± 15.80	46.00 ± 18.13	55.35 ± 16.42	41.63 ± 9.43	51.38 ± 13.82	60.04 ± 17.29	71.96 ± 7.83	60.08 ± 14.30	54.5 ± 16.1
Male	430	8	95	30	56	28	33	11	691
Female	274	6	43	16	28	19	21	2	409
Total patient number	704	14	138	46	84	47	54	13	1100
Scar/viability	149	2	48	39	15	6	0	7	266
Amyloid	37	0	0	0	1	2	0	0	40
HCM	115	1	16	0	8	8	0	0	148
Other cardiomyopathy	150	10	49	2	42	13	0	5	271
Mass	20	0	0	0	0	1	0	0	21
Congenital	9	0	2	0	2	3	0	0	16
Pericardial disease	27	0	1	1	1	0	0	0	30
Myocarditis/sarcoid	136	0	14	4	13	9	0	1	177
Other	61	1	8	0	2	5	54	0	131
Total study number	704	14	138	46	84	47	90	13	1136
Total studies per centre	718		268			47	103		1136
Training	491		180			33	-		704
Validation	122		45			8	-		175
Testing	105		43			6	103		257

^*^Dotarem

^**^Gadovist

For each study, a median (range) of 34 (9–100) frames was obtained in the TI scout series with median (range) inversion time 477.5 (231.0–1935.0) ms and median (range) temporal resolution 23.0 (8.8–45.3) ms. Images with the highest temporal resolution were acquired at centre 3 with median (range) of 16.7 (8.8–27.0) ms, with lower temporal resolution for one centre 2 system with median (range) 38.8 (23.3–45.3) ms. Images were retrieved in Digital Imaging and Communications in Medicine (DICOM) [16] format and anonymised using Python code.

A portion of the dataset (427/1100 subjects, detailed in Supplementary Materials) overlaps with previously published work, where the aim was to automate sorting of various cardiac MRI sequences, unrelated to the purpose of the current study [17].

### Ground truth

The image with optimal myocardial nulling (the null point) was selected from the TI scout series by a radiologist with 17 years’ experience with cardiac MRI (R.P.L., SCMR level 3 equivalent), and a radiology trainee (E.C.) with 2 years’ experience with cardiac MRI interpretation, following training sessions prior to independent labelling. A subset of centre 1–3 data (327/1033, 31.7%) was interpreted independently by both readers, with agreement assessed prior to further data labelling, and differences subsequently resolved in consensus for ground truth labels. As there was excellent agreement between independent readers (Spearman correlation 0.94), the remaining 706 centre 1–3 cases were labelled by the radiology trainee alone. Trainee-labelled data was used solely for model training, and the holdout test set from centres 1 to 3 used only consensus data. All centre 4 data was labelled by the expert radiologist for external validation of the final model.

### Data elements

**Fig. 1:**
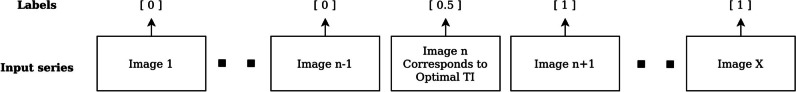
Model inputs and labels. Each image frame in a TI scout series is labelled, with the optimal TI given a soft label of 0.5, early images labelled with a 0 and late images labelled as 1. The model is trained to predict image *n* and then extract the corresponding inversion time for that image from its DICOM metadata

Model input was left ventricular mid short-axis TI scout images. The outcome variable for model development was the index of the optimal TI image. Each frame of the series was given a binary class—before (labelled 0) or after the null point (labelled 1). A soft label of 0.5 was given to the null point frame. Through a sequence-to-sequence approach, the model receives frames individually in a recurrent fashion, and then outputs the probability for the respective frames (Fig. 1). The transition point of these intermediary values is used to derive the outcome variable, i.e. for prediction, the image frame with the label closest to 0.5 is considered the optimal image (detailed under “Model design”).

### Data pre-processing

Several oversampling techniques were utilised during the training phase to help the model learn from underrepresented data as well as critical frames corresponding to the null point (further explained in Supplementary Materials—Data Preprocessing).

**Fig. 2:**
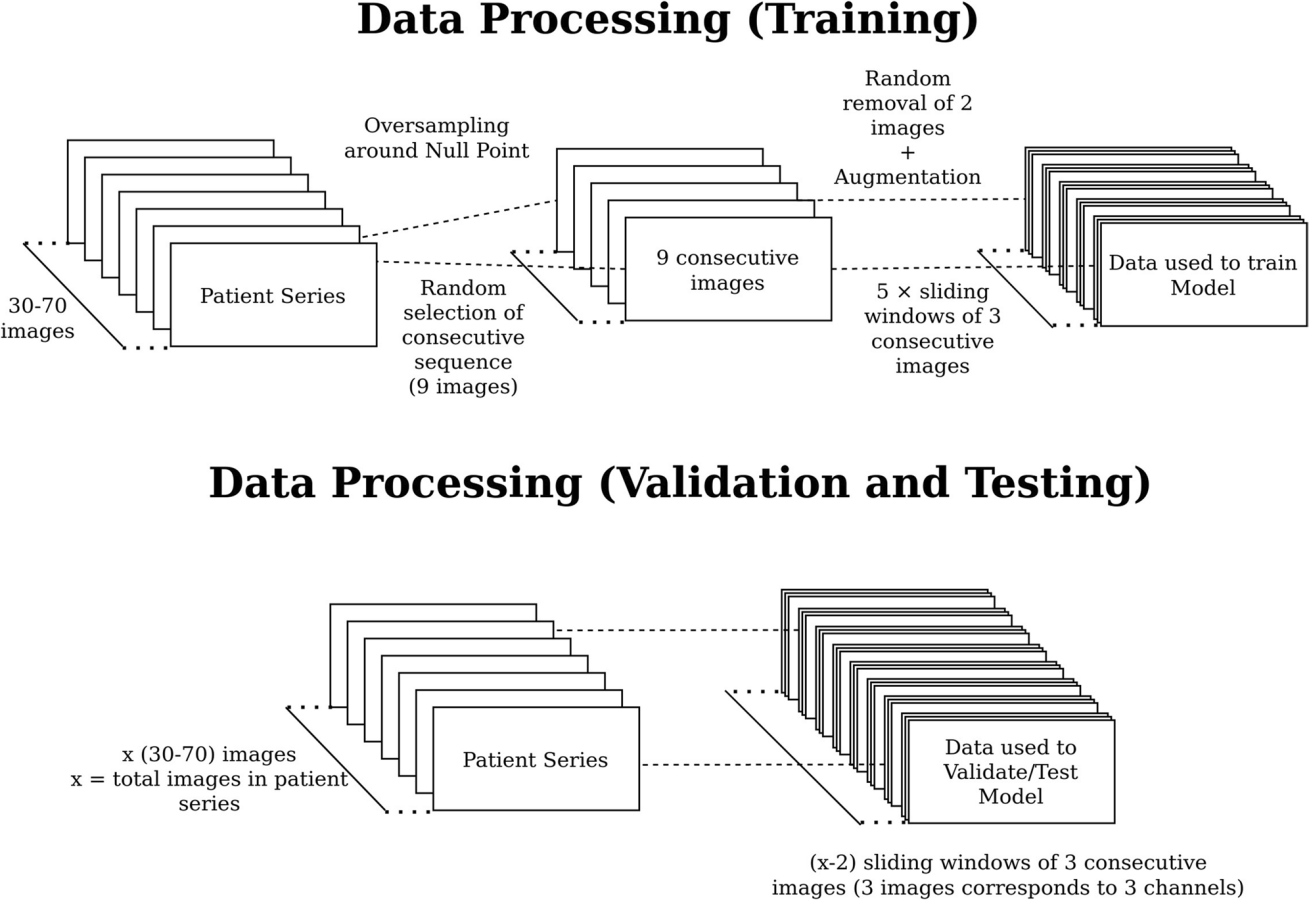
Data pre-processing. To provide consistent input for model training, nine consecutive images from each individual series were sampled, favouring frames near the labelled optimal TI. Following resizing and numerical normalisation, two images from the set of nine were randomly removed, and five sliding windows of three consecutive images were extracted for input. During the validation and testing phases, all images from each TI scout series were passed as is in groups of three consecutive images to the model, without oversampling or splicing

For every epoch during training, a splice of nine consecutive frames was sampled from each series. Images were standardised with global mean and variance, and then resized to 256 × 256 resolution through interpolation. Two frames were randomly removed from each nine-frame sequence to emulate varying temporal resolutions used in scout acquisition protocols across centres and magnet systems. The remaining seven frames were then grouped with their neighbouring frames (referred to below as a window), such that there were five sets of three adjacent frames across five timesteps, with oversampling around the null point when myocardial signal is suppressed (Fig. 2; additional details in Supplementary Materials).

During the testing and validation and testing phases, all images from each TI scout series were passed as is in groups of three consecutive images to the model, without oversampling or splicing (Fig. 2).

### Model design

**Fig. 3:**
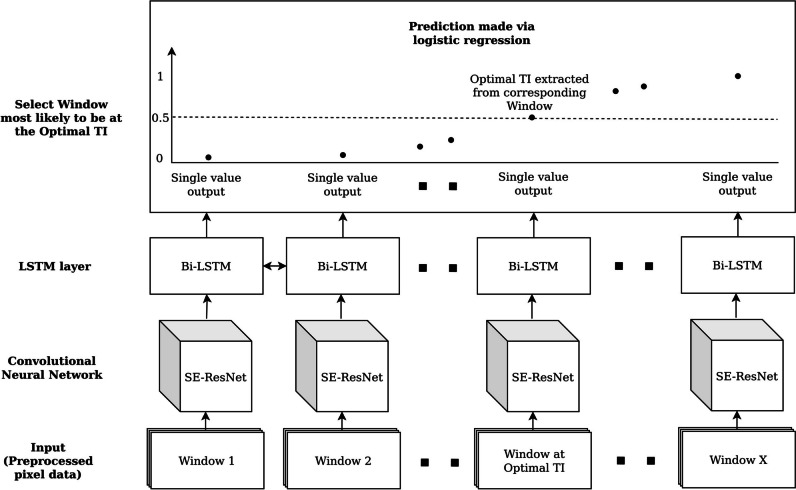
Model architecture and pipeline. Beginning with the input (bottom of figure) three images per window: (1) the CNN feature extractor receives the individual three-channel arrays obtained from the series (SE-ResNet given as an example); (2) pooled features from the CNN are passed into the bidirectional LSTM with logistic regression deployed on each timestep; (3) the window with an output probability closest to 0.5 is selected for prediction, with TI value of its centre frame returned as the predicted TI value

The model design consisted of two parts: a feature extraction CNN, and a bidirectional LSTM network (Fig. 3).

The CNN first iterates through all windows of a given series, where the channels correspond to the earliest, middle and latest frames within a given window, and thereby help identify both spatial features and temporal relationships between neighbouring frames (e.g. change in intensity). Three CNN architectures were evaluated—VGG16 [18], ResNet18 [19] and Squeeze-and-Excitation [20] SE-ResNet18, a modified ResNet architecture utilising Squeeze-and-Excitation blocks to perform channel-wise feature recalibration and enhance channel interdependencies. The CNN compresses features into a feature array with a dimension of 1024, to produce embedded representations of the windows.

The bidirectional LSTM network was then used to capture global temporal information beyond neighbouring frames. A final dense layer performs logistic regression and outputs a sequence of probabilities, where each value corresponds to a window. The predicted optimal TI was extracted from DICOM metadata (“InversionTime” for GE and “TriggerTime” for Siemens and Philips images) of the centre image of the window whose probability was closest to 0.5.

### Data partitioning

A total of 154 of 1033 samples (14.9%) from centres 1 to 3 were held out for testing. The remainder were used for training and validation, using five-fold cross-validation with an 80/20 training/validation split. The best-performing model from five-fold cross-validation was selected as the final model, whose performance was tested on the holdout test set from centres 1 to 3.

### Model training

Training was performed on an Nvidia Tesla V100 virtual partition with 16 gigabytes of VRAM, using Python 3.9.6 and PyTorch 1.9.0. Details of other packages are provided in Supplementary Materials.

Data augmentation was performed using an open-source framework (MONAI [21]) to expose the model to more heterogeneous training data and improve generalisability. Grid search was performed to identify the most suitable learning rate, decay rate and batch size. The CNN-LSTM model was trained end-to-end using ADAM optimisation over 30 epochs with a batch size of 32 and weight decay of 1 × 10^−4^. A differential learning rate was utilised, with an initial learning rate of 1.5 × 10^−2^ for the feature extractor and 3 × 10^−3^ for the LSTM. A weighted Binary Cross-Entropy loss function was used. For the loss of each sample, larger weights were applied around the null point:

$${{\text{weight}}}_{i}={\text{min}}({\text{abs}}\left({{{\text{log}}}_{e}\left(\frac{{T}_{i}}{{T}_{{\text{optimal}}}}\right)}^{-1}\right), 3)$$

where *T*_*i*_ is the inversion time at window *i*.

Detailed description of augmentations employed, model details including hyperparameters and the weighted loss function are provided in Supplementary Materials.

## Model evaluation

### Metrics

Model performance was evaluated with mean absolute error (MAE), accuracy (defined as model selection within 50 ms of ground truth, based on degree of variability of TI scout series of differing temporal resolutions and typical temporal resolution of cardiac MR sequences) [3], Lin’s concordance correlation coefficient (LCCC) and Bland–Altman analysis. Reduced major axis regression (RMAR) was also used to assess fixed and proportional bias of the predictions, with the latter not assessed with Bland–Altman analysis [22]. RMAR accounts for potential errors in both prediction and ground truth, as opposed to linear regression, which only incorporates prediction error. In this study, MAE was prioritised for model selection, as it most directly reflects the discrepancy between predicted inversion time and ground truth. Each metric was selected to reflect an aspect of model performance but may not completely correlate, as optimising for one metric may negatively impact others.

Inter-reader agreement on the holdout test set was also assessed with the same metrics for direct comparison. Subset analysis of model performance was carried out for individual centres, vendors and field strengths.

### Visualisations

2D histograms of prediction and ground truth distributions were created to visualise concordance between predicted inversion time obtained from the model and ground truth in 25-ms intervals. This bin size was selected to adequately display the density information of these distributions. Saliency maps were also employed to assist with model interpretability, further detailed in Supplementary Materials [23].

### External data

To assess generalisability, the final model was tested on all centre 4 data from two vendors (*n* = 103), previously unseen by the model during training and validation.

## Results

### Model evaluation

**Fig. 4:**
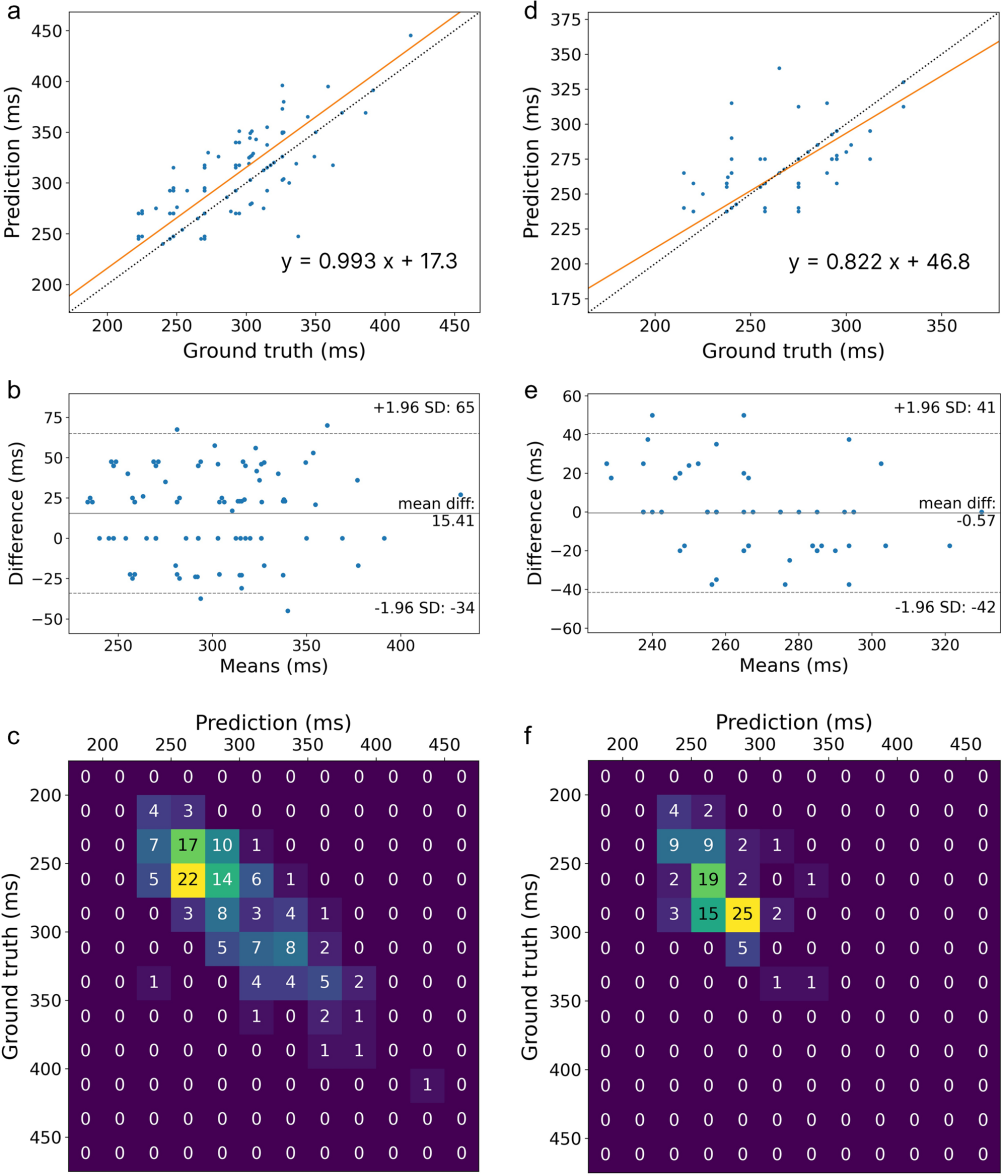
**a** Reduced major axis regression of holdout test data from centres 1 to 3 (*x*-axis is the ground truth inversion time, *y*-axis is the predicted inversion time, the dashed line represents the line of perfect concordance and the orange line the regression line), indicating a small fixed bias (tendency to overestimate inversion time). **b** Bland–Altman analysis of holdout test data from centres 1 to 3, showing a tendency to overestimate inversion time, consistent with RMAR. **c** 2D histogram of holdout test data from centres 1 to 3. Note that given the continuous nature of inversion time, the diagonal of histogram does not reflect the actual number of samples with MAE less than 25 ms. **d** Reduced major axis regress of data from centre 4, used exclusively for testing. The dashed line represents the line of perfect concordance and the orange line the regression line, indicating a tendency to slightly overestimate optimal TI at low inversion times and underestimate at higher inversion times. **e** Bland–Altman analysis of data from centre 4, with minimal mean difference and 95% limits of agreement within 50 ms. **f** 2D histogram of external data from centre 4

The best-performing model was SE-Resnet-18, which produced accuracy of 96.1%, MAE 22.9 ms and LCCC 0.472 on the holdout test set from centres 1 to 3 (Table 2), with further details of model selection provided in Supplementary Materials. RMAR demonstrated no proportional bias and fixed bias of 17.3 ms, indicating a slight tendency to overestimate the optimal TI (Fig. 4a, b). 2D histogram also shows strong performance of the final model to predict the optimal TI (Fig. 4c), with most errors within 50 ms of ground truth optimal TI, erring more on the side of overestimation of optimal TI. For comparison, inter-reader agreement yielded LCCC of 0.91, with no major proportional bias and similar fixed bias of 16.2 ms. The model performed better on 1.5 T data (accuracy 97.8%) than limited 3 T data (accuracy 72.7%), when data from centres 1 and 2 (where imaging at both field strengths was available) was analysed. Details of inter-reader agreement and subanalysis of model performance at different field strengths are provided in Supplementary Materials. Distribution of errors by centre and vendor is summarised in Table 3.

**Table 2:** Final model performance across different centres stratified by vendor on the holdout test set and external dataset

		Accuracy (% within 50 ms)	Mean absolute error (ms)	Mean squared error (ms^2^)	Lin’s concordance correlation coefficient	RMAR slope	RMAR intercept	BAA mean and 95% limits of agreement (ms)
Holdout test data	Centre 1—Siemens	97.1	22.7	883	0.421	0.93	34.6	16.17 (− 33, 65)
	Centre 2—Philips	91.7	23.3	870	0.538	1.33	-91.9	12.28 (− 40, 65)
	Centre 2—Siemens	100	27.5	1060	0.508	0.79	73.8	16.79 (− 38, 72)
	Centre 3—GE	100	19.3	596	0.925	1.01	16.2	19.25 (− 10, 49)
	Centres 1–3 combined	96.1	22.9	877	0.472	0.99	17.3	15.41 (− 34, 65)
External test data	Centre 4—Siemens	100	11.5	275	0.707	0.78	55.4	− 4.81 (− 36, 26)
	Centre 4—Philips	84.6	32.6	1580	0.325	1.25	-31.8	28.77 (− 25, 83)
	Centre 4 combined	97.3	15.2	502	0.641	0.82	46.8	− 0.57 (− 42, 41)

*RMAR* reduced major axis regression, *BAA* Bland-Altman analysis

**Table 3:** Predicted frames relative to ground truth by centre and vendor for holdout and external test sets, with same, underestimated and overestimated index frame results provided. More TI predictions were overestimated in the holdout test set (median 25 ms), whereas these were a minority in the external dataset, with greater proportion of correct predictions compared to the holdout test set. *MAE* mean absolute error, *IQR* interquartile range

	Same		Underestimated		Overestimated	
			Number of examinations	Median MAE (IQR) ms	Number of examinations	Median MAE (IQR) ms
Holdout test data	Centre 1—Siemens	33	11	22.5	61	25.0
				(2.5)		(22.5)
	Centre 2—Philips	9	9	23.0	18	30.0
				(7.0)		(23.8)
	Centre 2—Siemens	2	1	37.5	4	40.0
				(0.0)		(1.25)
	Centre 3—GE	2	0	-	4	26.5
						(6.0)
	Centres 1–3 combined	46	21	23.0	87	25.0
		(29.9%)	(13.6%)	(2.5)	(56.5%)	(22.5)
External test data	Centre 4— Siemens	42	35	17.5	13	20.0
				(2.5)		(7.5)
	Centre 4—Philips	2	1	25.0	10	25.0
				(0.0)		(25.0)
	Centre 4 combined	44	36	17.5	23	25.0
		(42.7%)	(35.0%)	(2.5)	(22.3%)	(16.3)

**Fig. 5:**
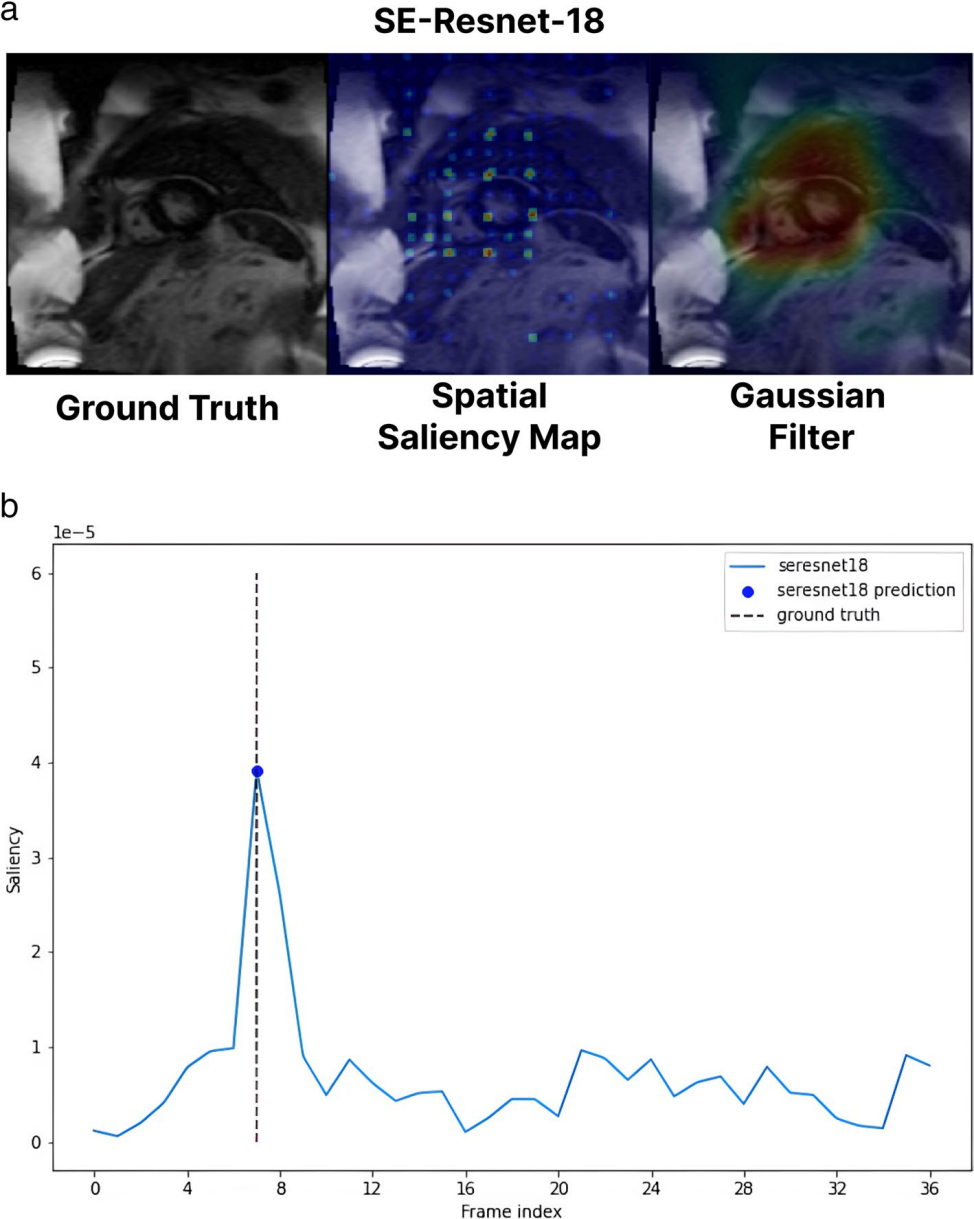
**a** Spatial saliency maps created by passing a given scout sequence to the final model (SE-Resnet18-LSTM), with the ground truth image (frame 7, left image) provided for comparison. These maps demonstrate attention to myocardium and surrounding structures. A Gaussian filter (right image) was applied to the map to help visualise residual connections, with the spatial saliency map demonstrating a grid-like pattern (centre image). **b** Temporal saliency of the final model showing strong model attention at the predicted null point at frame 7 (defined as average intensity over the whole saliency image for each frame)

Spatial saliency heatmaps of the model demonstrated a patchy grid-like pattern with attention primarily in the heart region (Fig. 5a). Attention was also present in surrounding tissues including the lung and intra-abdominal organs. The model paid little attention to aliasing artefacts in the image peripheries. The temporal saliency plot displayed strong attention in frames near the predicted null point (Fig. 5b).

### External data performance

The final model achieved accuracy of 97.3% on external data from centre 4, with MAE 15.2 ms and LCCC 0.641 (Table 2). RMAR demonstrated a predominantly proportional bias, with a tendency of the model to overestimate at low TIs and underestimate optimal TI at higher TIs (Fig. 4d, e). Results by centre and vendor are summarised in Table 3.

**Fig. 6:**
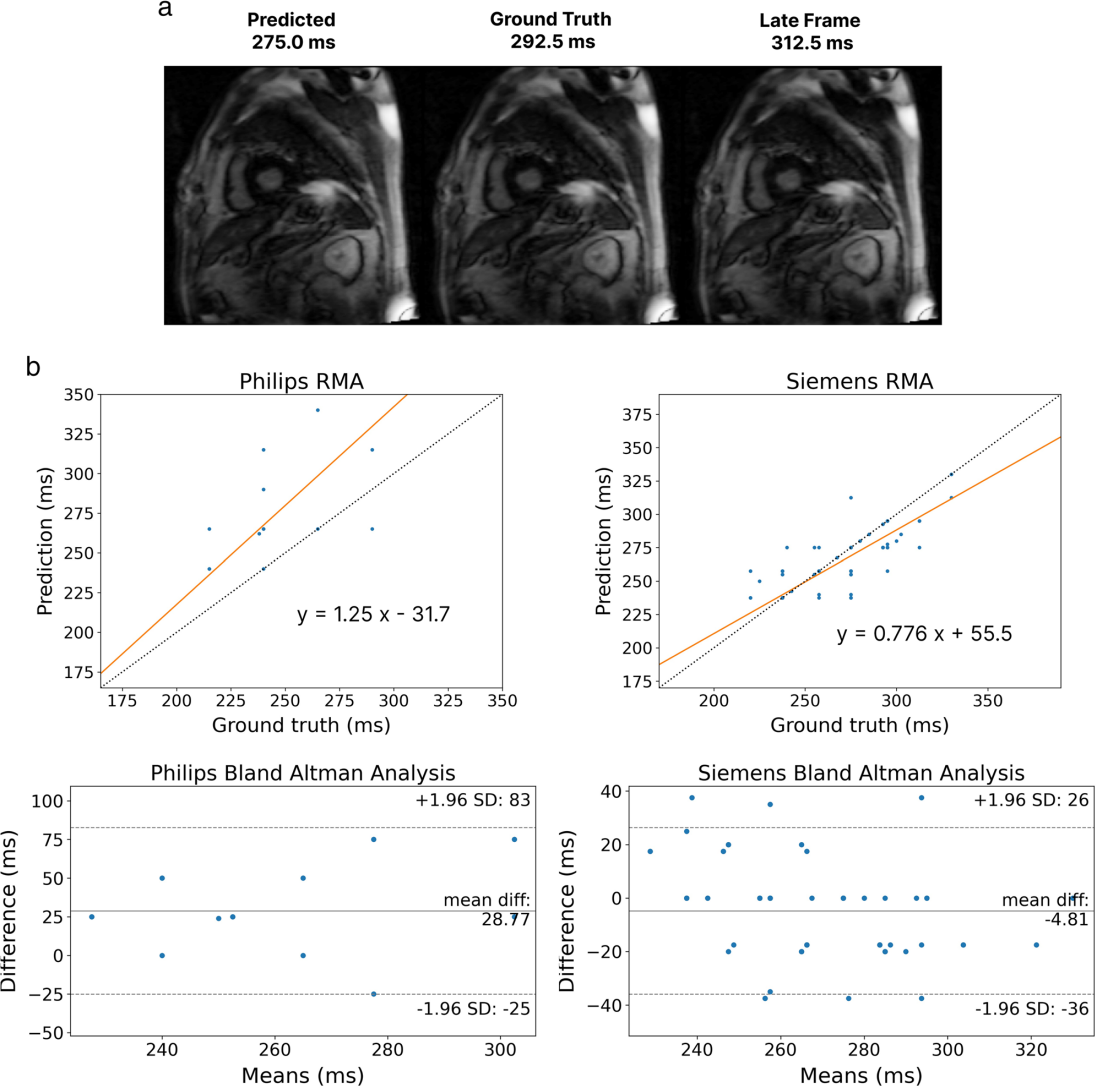
**a** The optimal TI transition point of a case from centre 4; in this example, the model underestimates the optimal time inversion by one frame. Visually, these images appear very similar. **b** Reduced major axis regression and Bland–Altman analysis of centre 4 data by scanner model (dashed line in RMAR represents the line of perfect concordance and the orange line the regression line)

The model achieved 100% accuracy within 50 ms for images from one vendor (Siemens) with a tendency to underestimate the optimal TI. Underestimations were within 50 ms of ground truth (35/90 optimal TIs underestimated, median ± IQR 17.5 ± 2.5 ms), with little discernible difference between ground truth and prediction on image review (Fig. 6a). There was 84.6% accuracy for images from the other vendor in the centre 4 dataset (Phillips), with a tendency to overestimate TI (10/13 optimal TIs overestimated, 2/13 by greater than 50 ms of the optimal TI, median ± IQR 25.0 ± 25.0 ms) (Fig. 6b). Subanalysis of field strength performance again yielded high accuracy for 1.5 T data for the external test set (91.1%), with 84.6% accuracy observed for 3 T data (further detail in Supplementary Materials).

The final model is made available at this site: https://github.com/AH-AI-in-Radiology/Cardiac_MRI_TI.

## Discussion

We have developed a serial CNN-RNN approach that can select optimal TI from multicentre multi-vendor MRI TI scout imaging with high accuracy. The potential benefit of an automated approach to TI selection is a method that is 100% reproducible, i.e. given the same input, the model will predict the same TI every time. Through reliable optimal TI selection, it could potentially assist with more accurate diagnosis, obviating poor or inconsistent nulling of normal myocardium that may occur for inexperienced or time-pressured operators. Our model was able to discern the transition from early to late frames, incorporating both spatial and temporal features. Although the RNN component of our approach was fixed as a LSTM, three CNN feature extractors were compared, VGG16 [18], Resnet18 [19] and SE-Resnet-18. The model containing a SE-Resnet-18 CNN achieved the best results, likely due to Squeeze and Excite Blocks. These blocks enable CNNs to perform dynamic channel-wise feature recalibration [20]. We postulate this allowed our model to extract a more discriminative set of features from a temporal series of images at the CNN level. Saliency mapping demonstrated model attention for both spatial (morphologic) and temporal features in predicting optimal TI.

The small (17.3 ms) overestimation of predicted optimal TI in centre 1–3 test data was uniform across TI (i.e. fixed bias). This may relate to the weighted loss function, which consistently applies larger weights to frames after the null point. In practice, minor overestimation is an acceptable error, as selection of later TIs than the optimal TI will still provide late gadolinium–enhanced imaging images where abnormal myocardium should still appear brighter than normal myocardium. Underestimation is potentially more problematic, as the relationship between normal and abnormal myocardium may be inverted if magnitude images alone are relied upon, with a more negative impact on diagnostic accuracy [24]. However, the underestimation observed in our external test data remained within 50 ms of ground truth, with similar visual appearance of predicted and ground truth images.

Poorer performance was noted for Philips data from centres 2 and 4. We postulate acquisition differences as a potential cause. This includes differences in the pulse sequence used to obtain TI scout imaging (balanced steady-state free precession for Siemens systems versus spoiled gradient echo or ultrafast gradient echo images from Philips and GE systems in our cohort), impacting tissue contrast and signal-to-noise. Temporal resolution likely also impacted accuracy, as systems with poorer temporal resolution/fewer frames were likely penalised in terms of accuracy, since true optimal TI is more likely to lie between frames. This is exemplified by the comparatively higher MAE for test set data from centre 2 (median temporal resolution 38.8 ms), and low MAE for centre 3 (median temporal resolution 16.7 ms). However, this was difficult to confirm in external test data which was predominantly obtained from a single-vendor system.

However, high model accuracy for the external data was observed. The heterogeneity of training data from various vendors may have contributed to its performance. The relative homogeneity of the centre 4 dataset (indication of aortic stenosis in the majority) may have contributed to the high observed accuracy. High prevalence of left ventricular hypertrophy in this disease might minimise difficulties with partial voluming of the LV myocardium that can be encountered in patients with low myocardial mass. However, we observed overestimation of optimal TI at lower TIs and underestimation at higher TI on centre 4 data, largely driven by results from the Siemens magnet. However, 101/103 (98.1%) predictions fell within 50 ms of the true optimal TI, and the degree of underestimation (potentially more problematic for late gadolinium–enhanced imaging) was small (all < 50 ms), with little visual difference in predicted versus ground truth imaging in these cases. Underlying pathology (with known prolongation of T1 myocardial values in aortic stenosis [25]), acquisition parameters, contrast type [26], dosage and timing of TI scout acquisition post contrast administration may all have influenced our results, highlighting the real-world variability of the TI scout sequence used between institutions.

Whilst Bahrami et al previously explored automatic detection of optimal TI with CNN and LSTM in a single-institution and single-vendor setting [14], we have developed a model using multicentre data at two field strengths that was able to accurately predict optimal TI for previously unseen data from two vendors from a centre not used for model training. We have shown that prediction of optimal TI is possible from pixel data alone, even for heterogeneous imaging acquired with different pulse sequences, at different field strengths and varying in terms of contrast type, dose and timing protocols. Additionally, we developed a distinct architecture which reduced the parameter counts substantially for improved training efficiency (from over 400 million to 32,258,625). A next step is implementation of such a model into clinical practice, as recently described by Ohta et al in a single-institution, single-vendor study utilising a VGG-19 model to aid TI selection in real time with a smartphone [27].

A limitation of the study was the unbalanced data distribution between centres and magnet systems. Centre 3 data was underrepresented, with only 6 datapoints in the holdout test set, and no representation of the centre 3 vendor in the external dataset. External test data was mainly from a single vendor with a relatively homogeneous patient population, which may bias model performance. Vendor-specific performance should therefore be interpreted with caution. 3-Tesla (3 T) scanner data was also underrepresented. Notably, only 5.6% (49/879) of training and validation data from centres 1 to 3 were from 3 T, probably accounting for poorer model performance on 3 T data. The model’s performance on patients with amyloid was not adequately assessed, with relatively few patients referred for this clinical indication. In clinical practice, optimal TI is difficult to determine in this population, but the TI scout itself can assist with diagnosis due to abnormal contrast kinetics [28]. Variable temporal resolution of the scout sequence in our data likely influenced model training and accuracy, particularly if the ideal TI lies between two discrete samples, impacting series with poorer temporal resolution. However, our dataset reflects heterogeneity of real-world clinical imaging.

We have developed a model capable of automatic detection of optimal TI with high accuracy to within 50 ms of optimal myocardial nulling that can be employed upon a range of sequences obtained from different institutions and vendors. We also share our model with the scientific community, to encourage further development or further testing on real-world data. With refinement, it could assist with efficient and reproducible TI selection for LGE imaging in cardiac MRI and could ultimately be incorporated into a fully automated clinical workflow.

### Supplementary Information

Below is the link to the electronic supplementary material.

Supplementary file1 (PDF 552 KB)

## Abbreviations

ADAM Optimiser: Adaptive Moment Estimation
CNN: Convolutional neural network
DICOM: Digital Imaging and Communications in Medicine
DL: Deep learning
LCCC: Lin’s concordance correlation coefficient
LGE: Late gadolinium–enhanced imaging
LSTM: Long short-term memory
LV: Left ventricular
MAE: Mean absolute error
MONAI: Medical Open Network for Artificial Intelligence
MRI: Magnetic resonance imaging
ResNet: Residual neural network
RMAR: Reduced major axis regression
SCMR: Society of cardiovascular magnetic resonance
TI: Time inversion
VGG: Visual Geometry Group

## Declarations

### Guarantor

The scientific guarantor of this publication is Ruth P. Lim.

### Statistics and biometry

One of the authors has significant statistical expertise (Professor L Churilov).

### Study subjects or cohorts overlap

Some study subjects or cohorts have been previously reported in “CardiSort: a convolutional neural network for cross vendor automated sorting of cardiac MR images,” Eur Radiol 2022 Sep;32(9):5907-5920, in unrelated work. In the current study, 60/718 centre 1 subjects’, 268/268 centre 2 subjects’, 47/47 centre 3 subjects’ and 52/65 centre 4 subjects’ image data was used in the previous publication. However, note the very different purposes of the two studies, with the prior study’s purpose to purely sort different cardiac MRI sequences, and the current work focusing upon one single sequence and automated prediction of optimal inversion time to facilitate scanning workflow. The majority of subjects in this current study have not previously been reported.

### Methodology

• retrospective

• cross-sectional study

• multicentre study
