# Supplementary material for: Automated inversion time selection for late gadolinium–enhanced cardiac magnetic resonance imaging
Source: Eur Radiol. 2024 Feb 10;34(9):5816–28. doi: 10.1007/s00330-024-10630-w (PMC11364710; doi:10.1007/s00330-024-10630-w)
Supplement: Supplementary file 1 — Supplementary file1 (PDF 552 KB) [file 330_2024_10630_MOESM1_ESM.pdf]

Supplementary Materials

<u>Scanner acquisition parameters.</u> Median (range) values for temporal resolution, frame number, inversion times provided.

Centre	Magnet System	N	Field Strength (T)	Base Sequence	Flip Angle (°)	Matrix	Slice thickness(mm)	TR (ms)	TE (ms)	Temporal resolution (ms)	Frame Number	Inversion Time (ms)	TI start (ms)	TI end (ms)	Time post injection (min)
1	Siemens - Avanto	704	1.5	bSSFP	30	192 x 78	8	2.6	1.1	23.0 (20.5-38.5)	34 (17-85)	760.0 (370.0-1935.0)	85.0 (75.0-87.5)	845.0 (0.0-2020.0)	8-15
	Siemens - Skyra	14	3	bSSFP	35	192 x 78	8	3.2	1.4	28.75 (28.5-28.75)	25 (22-36)	690.0 (602.5-1005.0)	100.0 (100.0-102.5)	790.0 (702.5-1105.0)	8-15
2	Philips - Achieva	46	3	Spoiled GRE	10	128 x 126	10	7.1	3.1	17.7 (16.6-34.6)	23 (11-41)	430.0 (231.0-671.0)	165 (160.0-168.0)	595.0 (391.0-836.0)	10-20
	Philips - Ingenia	138	1.5	Spoiled GRE	12	152 x 137	10	7.9	3.5	23.4 (17.8-39.0)	26 (16-56)	608.0 (352.0-1311.0)	163.0 (27.0-263.0)	747.0 (515.0-1339.0)	10-20
	Siemens - Aera	84	1.5	bSSFP	30	192x72	5	2.75	1.2	38.8 (23.3-45.3)	16 (9-37)	542.5 (350.0-1010.0)	80.0 (75.0-102.5)	617.5 (17.5-1087.5)	10-20
3	GE - Optima	47	1.5	Ultrafast GRE	5	128x128	10	4.39	2	16.7 (8.8-27.0)	60 (60-60)	983.3 (522.1-1594.6)	94.28 (92.9-95.5)	1077.61 (616.5-1688.8)	8-15
4	Siemens - Symphony	86	1.5	bSSFP	30	192 x 96	8	3.1	1.3	18.75 (18.5-25.0)	40 (22-100)	725.0 (392.5-1850.0)	87.5 (87.5-100.0)	798.75 (25.0-1937.5)	20
	Philips - Achieva	18	3	Ultrafast GRE	7	128 x 128	10	8	3.2	25.0 (24.1-25.0)	24 (14-28)	575.0 (325.0-650.0)	165.0 (165.0-165.0)	740.0 (490.0-815.0)	10-20

Subject Overlap:

There is some subject overlap with previously published work ("Cardisort: a convolutional neural network for cross vendor automated sorting of cardiac MR images"[1]), where the goal was simply to sort various cardiac MRI sequences. In the current study, 60/718 Centre 1 subjects, 268/268 Centre 2 subjects, 47/47 Centre 3 subjects and 52/65 Centre 4 subjects' image data was used in the previous publication. However, note the very different purposes of the 2 studies, with the prior study purpose purely to sort different cardiac MRI sequences, and the current work focusing upon a single imaging sequence (TI scout imaging) and automated prediction of optimal inversion time to facilitate scanning workflow, crucial for optimal myocardial nulling of late gadolinium enhanced imaging.

Inter-observer variability

The following table, together with reduced major axis regression and Bland-Altman analysis, demonstrate excellent agreement between the two independent readers on the hold out test set, with results from the final model included for comparison.

	Mean absolute difference (ms)	Lin's concordance correlation coefficient	RMAR slope	RMAR intercept	95% Limits of agreement (ms)
Inter-observer variability	4.99	0.913	0.94	16.2	-1.52<br>(-34, 31)
Final model	22.9	0.472	0.99	17.3	15.41<br>(-34, 65)

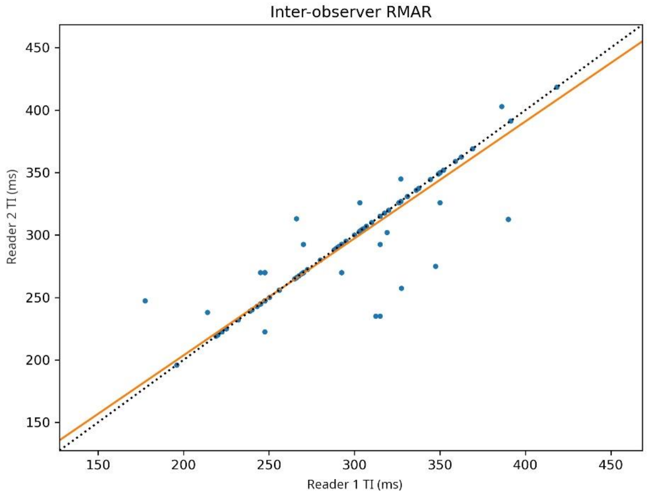

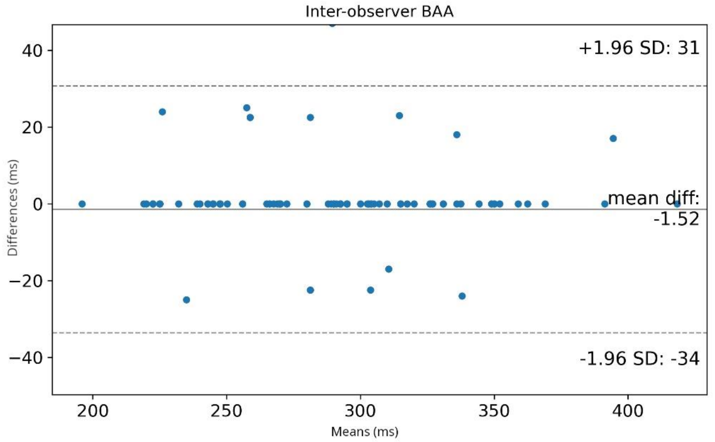

Data Preprocessing

Subset oversampling algorithm

As data was unevenly distributed across centres, scout series from under-represented centres were oversampled during training. The training set was first divided into subsets based on the centre and scanner vendor. At the end of every epoch, the sampling weights of poor-performing subsets were increased by comparing the mean absolute error (MAE) of the respective subsets against that of the entire training set to incrementally improve the model's performance on these subsets.

1. Each subset begins with the same sampling weight:

$$W_{vendor} = \frac{1}{4}$$

2. Errors are calculated for each subset individually:

$$(MAE_{Centre\ 1}, MAE_{Centre\ 2\ Siemens}, MAE_{Centre\ 2\ Philips}, MAE_{Centre\ 3})$$

3. The overall error is the average across all subsets:

$$MAE_{overall} = \frac{MAE_{Centre\ 1} + MAE_{Centre\ 2\ Siemens} + MAE_{Centre\ 2\ Philips} + MAE_{Centre\ 3}}{4}$$

4. The relative error for each vendor is given by the ratio:

$$R_{vendor} = \frac{MAE_{vendor}}{MAE_{overall}}$$

5. Sampling weights are updated after the epoch to bring the relative error closer to 1:

$$W_{vendor} \leftarrow W_{vendor} \cdot (alpha \cdot (R_{vendor} - 1) + 1)$$

where alpha determines the step size of update (0.5 was selected in our training). A relative error greater than 1 will result in an increased weight, and vice versa for values less than 1.

6. The weights are scaled to ensure the sum is 1:

$$W_{vendor} \leftarrow \frac{W_{vendor}}{\Sigma W_{vendor}}$$

Commented [LR1]: I don't see the equation that is meant to be here? Please also fix in Response to Reviewers doc

Commented [LR2]: ditto

Oversampling around the null points

Since the model outputs whether a frame is early or late, the null point serves as a decision boundary and is essential to an accurate prediction. As such, frames near the null point were oversampled to encourage the model to learn from these critical frames. After selecting a scout series from the training dataset, a short segment of nine consecutive frames were sampled from this series using a log-normal distribution centred around the null point (i.e. the index of the 5th frame was given by distribution with μ of $\log_e(TI_{optimal})$ and σ of 0.5). This distribution emphasises the earlier frames, where most variability in the scout series occur. It also mitigated the issue of series length variability in the data, where some series were considerably longer than others, resulting in large quantities of late frames.

If frames needed to be sampled outside the bounds of the original series (e.g. earlier than the earliest possible frame), these would be padded with the nearest frame. The σ of 0.5 was selected to minimise the need for padding while allowing the model to have adequate exposure of frames across various TIs.

Data augmentation parameters

Augmentations were selected by the radiologist to maximise variability while ensuring inversion time information was retained. The extremes were reviewed by the same radiologist to ensure that optimal inversion time remained recognisable

Data augmentation was performed using an open-source framework (MONAI [2]). Random composite geometric transformations were then applied to all images in the series (elastic, affine, rotation and zoom), followed by random intensity alterations (Gaussian smooth, K space spike noise, Gaussian noise). To improve the generalisability across multiple scanners, the intensity and contrast were randomly scaled. These augmentations were applied to every sequence sampled, using the same parameters for each image within the series. The following hyperparameters were used:

- Rand2DElastic: spacing 30.0, magnitude range 0.0 to 1.3
- RandAffine: shear range -0.8 to 0.8, scale range -0.5 to 0.5, translate range -40 to 40
- RandRotate: range x 3.14, range y 3.14
- RandZoom: min zoom 0.8, max zoom 1.2
- RandGaussianSmooth: sigma x 0.25 to 1.0, sigma y 0.25 to 1.0
- RandKSpaceSpikeNoise: intensity range 4 to 14.0
- RandGaussianNoise: mean 0.0, std 5.0
- RandScaleIntensity: factors -0.33 to 0.5
- RandAdjustContrast: gamma 0.5 to 4.5

Probability of 1 for all the above

Model Details

CNN Embedding Specifications

The dense layers of CNNs were removed, keeping only the convolutional component for feature extraction. For the two residual networks, Resnet 18 and SE-Resnet 18, the final global pooling layer was replaced with an adaptive average pooling layer, with an output resolution of 4 by 4, to partially retain spatial information.

The CNN-extracted features were further compressed to produce embedded representations of the windows, each with a dimensionality of 1024. This reduction in dimensionality served to reduce parameter count (e.g. from 53,227,073 to 32,258,625 for SE-ResNet18) and overfitting. This is achieved with a dropout layer (dropout rate 0.5), a linear layer (1024 output units), batch normalisation, and a ReLU activation layer.

LSTM details

The LSTM contained 2 layers, each with 512 hidden states. Dropout layers with rate of 0.2 were applied before, within and after the LSTM network.

Model Hyperparameters

Grid search was performed to identify the suitable learning rate, decay rate and batch size. A higher learning rate was used in earlier portions of the model as it receives all windows in the sequence, thereby having an effective batch size of 320 (5 windows per sequence with 64 sequences in a batch). Scaling learning rate proportionally may reduce generalisation gap[3]. The learning rates were decayed in a stepwise manner at epoch 15 and 25, with a gamma of 0.3. These epochs were selected from values obtained from a reduce-on-plateau scheduler and were later pre-set to improve the reproducibility of the training process.

Weighted Binary Cross-Entropy loss

It was noted that a low loss may not translate to a high accuracy, which relies primarily on predictions near the null point. Therefore, larger weights were given to windows closest to the optimal inversion time. The weight at window i is described by the following:

$$weight_i = min(abs(log_e(\frac{T_i}{T_{optimal}})^{-1}), 3)$$

Where TI is the inversion time at window i. An upper bound of 3 was applied to avoid numerical instability.

Modified saliency map algorithm

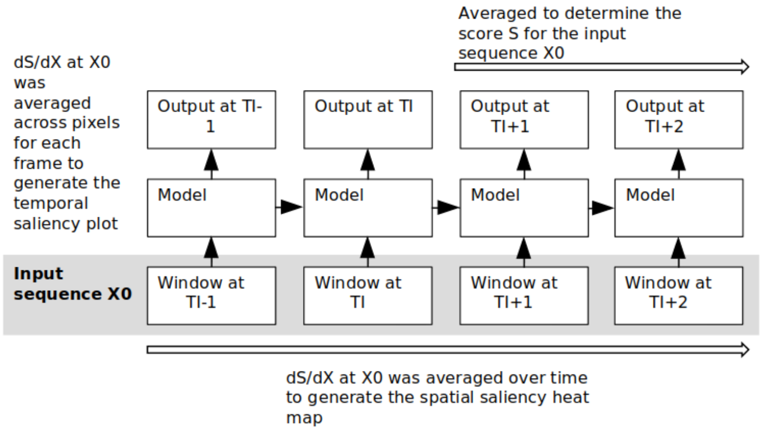

We employed a modified technique such that both temporal and spatial activations could be visualised. Specifically, all outputs after the predicted null point were averaged to create a single score for the given input sequence. Furthermore, the derivative of this score was averaged across the spatial and temporal dimensions to assess saliency in these axes separately. Heatmaps with Gaussian smoothing were produced to assist interpretation of visualisations for models with residual connections (SE-Resnet and Resnet).

Model Selection

The model containing SE-Resnet-18 as the feature extractor exhibited the lowest median(range) MAE 19.9(19.1-22.7) ms, compared to Resnet-18 with 21.8(19.9-32.0)ms, and VGG16 with 25.6(23.3-40.4)ms. It also had a median (range) accuracy of 96.5 (94.2-97.7)% on 5-fold

cross-validation, exceeding the performance of both Resnet-18 (93.0%, range 86.5-96.5%) and VGG16-based models (91.2%, range 80.7-93.6%). The best performing fold of SE-Resnet-18 was selected as the final model.

The 5-fold cross validation performance of these three models are presented in the following table. Median values are presented with range in parentheses. RMAR = reduced major axis regression, providing assessment of both proportional (slope) and fixed (intercept) bias. BAA = Bland-Altman analysis

	Accuracy (% within 50 ms)	Mean absolute error (ms)	Mean squared error (ms^2)	Lin's concordance correlation coefficient	RMAR slope	RMAR intercept	BAA mean and 95% limits of agreement (ms)
SE-Resnet-18	96.5 (94.2-97.7)	19.9 (19.1-22.7)	808 (730-1533)	0.613 (0.559-0.755)	1.28 (1.01-1.35)	-84.1 (-110.6 - -9.2)	4.89 (-54, 64)
Resnet-18	93.0 (86.5-96.5)	21.8 (19.9-32.0)	994 (756-1592)	0.579 (0.454-0.748)	1.16 (1.02-1.41)	-56.9 (-119.9 - 2.6)	6.48 (-59, 72)
VGG16	91.2 (80.7-93.6)	25.6 (23.3-40.4)	1464 (1014-2160)	0.537 (0.393-0.640)	1.09 (1.03-1.22)	-4.6 (-34.6 - 6.6)	-19.45 (-86, 47)

Model Visualisation Comparison

In models that contained residual connections (SE-Resnet and Resnet), spatial saliency appeared patchy in an almost grid-like pattern. This was not present in the VGG-based architecture, whose heatmaps retained fine granularity with attention to the left ventricular myocardium and

adjacent structures. The temporal saliency plot for the residual-based networks displayed strong attention in frames near the predicted null point. In comparison, the VGG-based architecture had most attention towards the later frames. Gaussian filter was applied to the map to help visualise models with residual connections.

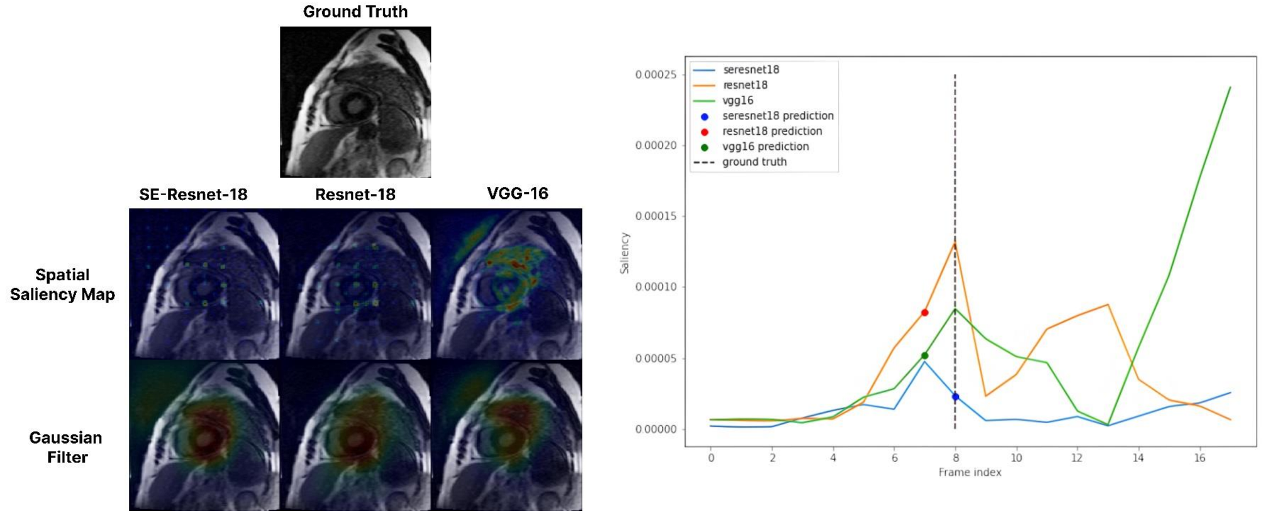

<u>5-fold cross validation performance for various techniques utilised for the best performing model architecture incorporating SE-Resnet-18</u>

The following table demonstrates the incremental improvements in accuracy associated with techniques utilised in training the best performing model architecture (i.e. SE-Resnet-18). The model was retrained with each of the following techniques removed. The median performance of 5-fold cross validation is provided in table below:

	%Accuracy within 50 ms (median and max/min values)	Mean absolute error (ms)	Mean squared error (ms^2)	Lin's concordance correlation coefficient	RMAR slope	RMAR intercept
**All techniques**	96.5 (94.2-97.7)	19.9	807.9	0.61	0.78	65.7
**Without weighted loss function**	95.3 (90.6-96.5)	21.9	892.2	0.62	0.77	73.2
**Same learning rate in CNN as RNN[1]**	95.3 (84.2-95.3)	21.2	936.0	0.63	0.89	15.09
**Without Random drop[2]**	95.3 (78.4-95.9)	22.5	930.9	0.58	0.75	58.0
**No oversampling around the null points[3]**	94.7 (92.4-97.7)	21.8	955.8	0.56	0.79	62.4
**No subset oversampling[4]**	94.7 (86.5-96.5)	22.3	970.0	0.61	0.75	80.8

1 – Using a learning rate of $3x10^{-3}$ throughout the model

2 – Without randomly removing 2 frames from the series (see Methods – Data Pre-processing)

3 – As described in Appendix 4 - Oversampling around the null points

4 – As described in Appendix 3 - Subset oversampling algorithm

Comparison of model performance between 3T and 1.5T testing data

The following table shows relatively poor performance on 3T-specific holdout and external test data when compared to those of 1.5T. This may be due to a combination of limited 3Ttraining data (5.6% of the training/validation data from Centres 1-3 were 3T) and 3T testing data. Note that there were only 11 and 13 testing instances for 3T from holdout and external test data respectively. Therefore, these results should be interpreted with caution. Please note Holdout test data from Centre 3 was excluded from this analysis as it contained only 1.5T sequences.

		Accuracy (% within 50ms)	Mean absolute error (ms)	Mean squared error (ms^2)	Lin's concordance correlation coefficient	RMAR slope	RMAR intercept
Holdout test data - Centre 1 and 2	1.5 T (N=137)	97.8	22.7	865	0.604	0.97	24.2
	3 T (N=11)	72.7	27.9	1180	0.551	0.99	23.4
External test data	1.5 T (N=90)	91.1	11.5	275	0.707	0.78	55.5
	3 T (N=13)	84.6	32.6	1580	0.325	1.25	-31.7

List of packages

- **python 3.9.6**
- **torch 1.9.0**

- **timm 0.5.0**
- **monai 0.6.0**
- **ray 1.10.0**
- **wandb 0.12.2**
- **numpy 1.21.2**
- **pandas 1.3.2**
- **statsmodels 0.13.2**

References

1. Lim RP, Kachel S, Villa AD, et al. CardiSort: a convolutional neural network for cross vendor automated sorting of cardiac MR images. *European radiology*. 2022;32(9):5907-5920.
2. Cardoso MJ, Li W, Brown R, et al. Monai: An open-source framework for deep learning in healthcare. *arXiv preprint arXiv:221102701*. 2022;
3. He F, Liu T, Tao D. Control batch size and learning rate to generalize well: Theoretical and empirical evidence. *Advances in Neural Information Processing Systems*. 2019;32
